# Regional Assessment of Urban Impacts on Landcover and Open Space Finds a Smart Urban Growth Policy Performs Little Better than Business as Usual

**DOI:** 10.1371/journal.pone.0065258

**Published:** 2013-06-05

**Authors:** James H. Thorne, Maria J. Santos, Jacquelyn H. Bjorkman

**Affiliations:** 1 Information Center for the Environment, University of California Davis, Davis, California, United States of America; 2 Spatial History Project and Bill Lane Center for the American West, Stanford University, Stanford, California, United States of America; University of Florida, United States of America

## Abstract

Assessment of landscape change is critical for attainment of regional sustainability goals. Urban growth assessments are needed because over half the global population now lives in cities, which impact biodiversity, ecosystem structure and ecological processes. Open space protection is needed to preserve these attributes, and provide the resources humans need. The San Francisco Bay Area, California, is challenged to accommodate a population increase of 3.07 million while maintaining the region’s ecosystems and biodiversity. Our analysis of 9275 km^2^ in the Bay Area links historic trends for three measures: urban growth, protected open space, and landcover types over the last 70 years to future 2050 projections of urban growth and open space. Protected open space totaled 348 km^2^ (3.7% of the area) in 1940, and expanded to 2221 km^2^ (20.2%) currently. An additional 1038 km^2^ of protected open space is targeted (35.1%). Urban area historically increased from 396.5 km^2^ to 2239 km^2^ (24.1% of the area). Urban growth during this time mostly occurred at the expense of agricultural landscapes (62.9%) rather than natural vegetation. Smart Growth development has been advanced as a preferred alternative in many planning circles, but we found that it conserved only marginally more open space than Business-as-usual when using an urban growth model to portray policies for future urban growth. Scenarios to 2050 suggest urban development on non-urban lands of 1091, 956, or 179 km^2^, under Business-as-usual, Smart Growth and Infill policy growth scenarios, respectively. The Smart Growth policy converts 88% of natural lands and agriculture used by Business-as-usual, while Infill used only 40% of those lands. Given the historic rate of urban growth, 0.25%/year, and limited space available, the Infill scenario is recommended. While the data may differ, the use of an historic and future framework to track these three variables can be easily applied to other metropolitan areas.

## Introduction

Globally, over 50% of all humans live in urban areas [1]. To these 3.3 billion urban dwellers, are projected an additional 1.75 billion people by 2030 [2], a trend in both developing [1] and developed countries [3], [4]. The process of urbanization transforms natural landscapes and can have irreversible impacts to biodiversity, ecosystem structure, ecological processes, and agriculture [5], [6], [7], [8]. Extinction rates of native plants increase in urban areas [9], invasive plant species are found in higher concentrations than in the surrounding countryside [10], and a majority of studies found a decrease in vertebrate species richness associated with urban areas [11]. The rapid growth of cities has led to increasing research on urban ecology [7], research about urban impacts to natural ecosystems [12], and recognition of the need for regional planning that can integrate establishment of nature reserves and open space within expansion of urban areas [9], [13], [14].

The progression of urban growth and conservation of open space often do not co-occur, due to high monetary costs for the acquisition of open space parcels, the race to acquire land for development, and because developed areas are sometimes considered to have little conservation value [15], [16]. Nonetheless, urban areas can hold remnant populations of important species [12]. Parks within urban areas may serve as habitat, and these open space elements may make up or complement a protected area network [17]. Further, green spaces in metropolitan areas have been shown to improve the quality of life within the urban area by promoting and rebuilding connections between people and nature [12]. The varying uses of open space, and the competition for it by different users, points to the need for further study of the dynamics of urban growth and protection of open space from spatial and ecological perspectives.

We suggest the use of a simple regional assessment framework consisting of three elements that can be tracked through time: urban growth, protection of parks, trails and open space, and changes in vegetation type extents. This approach has the flexibility to include different time frames allowing for a continuum between historic trends and future projections of different growth scenarios. The framework permits a simple quantification of how well regions with urban centers have protected natural vegetation and open space. Spatial extents of historic and projected future urban areas can be contrasted with the extent of natural ecosystems lost, and with the establishment of open space on a time step basis. For future projections, the approach can be used to test the performance of different urban growth policies, such as the concept of Smart Growth [18], which advocates increased density of new urban construction.

As an application of this approach, we combined trends detected from historic data and future projections from an urban growth model to gain some perspective on the dynamics of urban growth, open space, and natural vegetation. We used the San Francisco Bay Area (Bay Area), California, USA, as a study system. The region has historically experienced both large population growth, and a long history of successive efforts to conserve open space [19]. We used the Bay Area to: (1) quantify changes in urban (developed) area extent, in parks, trails, and open space (Open Space), and in landcover types from 1940 to 2010; (2) assess the progress of conservation efforts relative to urban growth; (3) review future urban growth model outputs and evaluate the impact of three policy scenarios on current landcover types and on proposed future open space; and, (4) to test how well a Smart Growth policy performed relative to a Business-as-usual policy.

## Materials and Methods

### Study Area

**Figure 1:**
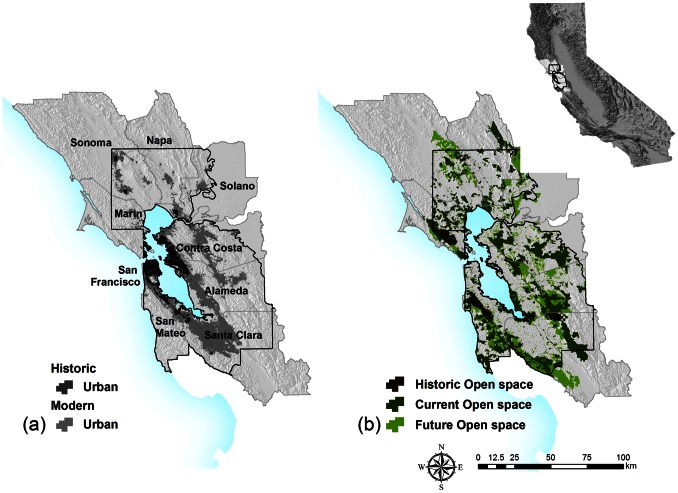
The San Francisco Bay Area, (a) including historic and current urban areas for Alameda, Contra Costa, Marin, Napa, San Francisco, San Mateo, Santa Clara, Solano, and Sonoma counties in California, USA; (b) historic, modern and future Open Space in the study area. The extent of this study is limited to 9275 km^2^ that were surveyed by the historic landcover mapping effort (inside the dark line).

Located in northern California, the San Francisco Bay Area has a Mediterranean climate with mild wet winters and dry summers and with summer fog in some areas. Native vegetation types in the region are predominantly oak and chaparral communities, large expanses of native grasslands, and mixed conifer and hardwood forests. The region’s nine-county population reached 7.15 million in 2005 from 1.73 million in 1940 [20], with projections to grow to 10.22 million by 2050 according to the Public Policy Institute of California (PPIC) [21]. Historic population growth and associated urban development co-occurred with the implementation of a network of parks and Open Space that covers about 20.2% of the study area (Figure 1). A 9275 km^2^ portion of the Bay Area was suitable for this analysis because of baseline historic landcover mapping surveyed between 1928 and 1936 (Figure 1) [22].

### Landcover

The United States Forest Service (USFS) conducted the first spatial inventory of California's forests in the 1930s, headed by Albert Wieslander [23], [24]. The Wieslander Vegetation Type Maps (VTMs) survey maps record dominant vegetation polygons (here called landcover), hand-drawn on United States Geological Survey (USGS) topographic maps by field crews who observed and recorded the patterns of vegetation. Surveyors recorded from one to nine species codes denoting the dominant plant species by rank order of cover within each polygon. The same surveyors worked for the project for over 10 years, and were highly experienced in vegetation mapping. They mapped ∼100,000 km^2^ of California. They surveyed an associated >16,000 vegetation plots, preserved over 25,000 voucher specimens, and took over 3,000 photographs, all of which are preserved at the University of California, Berkeley. Eleven VTM quadrangles were surveyed in the Bay Area from 1928–1936 covering 9275 km^2^, which we digitized to GIS (Figure 1 see methods in [24]). The species combinations identified in each VTM polygon were assigned to 27 California Wildlife Habitat Relationship (CWHR) landcover types [25]. CWHR is a simple classification scheme based on dominant plant species and physiognomy commonly used for resource management in California. While we did not conduct a formal accuracy assessment for the VTM maps due to lack of reference data, our crosswalk to assign species combinations to CWHR types was reviewed by a botanist from the USFS to verify, and less than one percent of polygon classifications were updated.

The Bay Area VTM maps have varying degrees of spatial accuracy depending on the topographic quadrangle and complexity of the underlying topography. We used Root Mean Square Error (RMSE) to measure the co-registration error of the historic and current landcover maps and to set the grid cell size for the subsequent analysis (following [26]). RMSE measures the spatial displacement (in meters) between two cells that should be collocated in the same geographical space [26]. RMSE value was a mean 76.3±22.3 m, so we selected a 100 m grid as the operational scale of the analysis, and resampled each map into this cell size. This had the effect of accounting for spatial inaccuracies in both maps, and of smoothing the finer spatial mapping in the modern maps.

The Bay Area Open Space Council created the Bay Area Upland Habitat Goals regional landcover map to support a plan for additional designated Open Space in the Bay Area called the Conservation Lands Network [27]. The BAUHG map is a compilation of the best available contemporary maps, which are predominantly based on 30 m satellite imagery, none older than 2006, and with the primary source being a USFS map called CalVeg (http://www.fs.fed.us/r5/rsl/projects/mapping/accuracy.shtml). The BAUHG map contains 20 CWHR landcover types [27]. Thematic accuracy is not published for the BAUHG map or its component maps. However the BAUHG map, was peer reviewed before its publication. We used the BAUHG map to represent current conditions, and resampled it using majority rule to the same 100 m grid frame.

Because each landcover map method identified habitats in slightly different ways, we assigned the CWHR types in the VTM and BAUHG maps to a simplified landcover system comprised of 12 types (Table 1), to ensure the maps were thematically comparable. For example, the historic map permits clear distinction of different oak woodland types, but the modern map in some cases, can only distinguish that it is a hardwood woodland, and the species remain unspecified. Our new classification includes four composite classes: Chaparral, Conifer-hardwood, Hardwood and Riparian. The Chaparral definition includes three CWHR types of inland chaparral: Mixed Chaparral, Chamise-Redshank (*Adenostoma fasciculatum* and *A. sparsifolium*) Chaparral, and Montane Chaparral; the Conifer-hardwood class included Douglas fir (*Pseudotsuga menziesii*), Ponderosa Pine (*Pinus ponderosa*), Redwood (*Sequoia sempervirens*) and Montane Hardwood-Conifer; the Hardwood class included Blue-oak (*Quercus douglasii*) Foothill-Pine (*Pinus sabiniana*), Blue Oak Woodland, Coastal Oak (*Q. agrifolia*) Woodland, Eucalyptus, Montane Hardwood and Valley Oak (*Q. lobata*) Woodland; while the Riparian class contains two CWHR types Montane Riparian and Valley Riparian. There were only 8 km^2^ of pure conifer, which were included with conifer-hardwood. We calculated a transition matrix from historic to modern on a per 100×100 m cell basis. This transition matrix documents the net change in recorded landcover types over the 70 year period.

**Table 1:** Area (km^2^) covered by each landcover class in each time period and projected urban growth within current vegetation types in the Bay Area.

	Historic (km^2^)	Modern (km^2^)	Difference	Convertedto urban	Projected urban –BAU (km^2^)	Projected urban –SG (km^2^)	Projected urban – IF (km^2^)
Agriculture	3222	543	−2679	1440	169	134	21
Grassland	2161	2763	601	257	584	536	107
Coastal Salt Marsh	182	111	−70	37	2	2	1
Coastal Chaparral	270	229	−41	10	21	16	1
Chaparral	673	667	−6	7	36	28	4
Hardwood	1796	1731	−65	71	236	202	29
Conifer-hardwood	246	606	361	0	30	29	8
Barren	46	42	−4	5	1	1	1
Riparian	2	41	39	0	5	4	2
Wetland/Riparian	14	42	28	4	1	1	1
Water	267	262	−5	56	5	4	3
Developed	396	2239	1843	*351*	*92*	*88*	*626*
Total	9275	9275	0	2239	1183	1044	804

Historical corresponds to 1940’s; current corresponds to 2006 and difference is current minus historical. Projected corresponds to 2050; BAU – Business-as-usual, SG – Smart-growth, and IF – Infill scenarios. Numbers in italics indicate urban re-development (i.e. an increase in density class).

### Urban Growth

We obtained historic urban extents from the VTM maps, and current extents from the BAUHG landcover map.

We used an urban growth model – UPlan [28], [29] – to project future urban growth in the Bay Area to 2050. UPlan is a rule-based model that uses a combination of demographic growth projections; government zoning that identifies where different types of human activities may occur; and geographic features weighted to be more or less attractive to spatially project urban growth, or masked to not build. UPlan projects where multiple densities of housing, commercial, and industrial development will occur into a 50 m grid of the study area. UPlan’s spatially explicit outputs can be made to simulate the spatial patterns of different policy scenarios by changing the number of residential units per area to simulate different urban densities, and by changing the attraction, discouragement, and masked (do not build) values of grid cells in different patterns.

UPlan outputs have been used to characterize policy impacts by overlaying them with other natural resource maps [30], [31], [32], [33]. We used human population growth projections on a county-by-county basis from the PPIC for 2050 [21], [34], in conjunction with county general development plans [35], and landscape features that attract or discourage urban growth (for details on model parameterization see Appendix SI). UPlan uses these inputs to spatially project new development for housing, commercial and industrial structures iteratively, placing each of the projected number of new units on the landscape into the grid cell with the highest attraction value, until all projected new growth is allocated. We ran UPlan for the Bay Area counties to the output year 2050, and simulated three growth scenarios: Business-as-usual, Smart Growth and Infill, by changing the housing densities used and the proportions of new growth assigned to different densities.

Business-as-usual represents a continuation of the current development policy, and used existing household and employment data for 2000, from the United States Census Bureau [36], [37], [38], Public Use Microdata Sample (PUMS), and the United States Census Transportation Planning Product (CTPP). We divided the projected housing needs for 2050 into four residential density classes according to the current proportions between them, to arrive at a total footprint (Appendix S1). The Business-as-usual scenario used the following residential classes: R20 (20 units/0.4 ha); R5 (5 units/0.4 ha); R1 (1 unit/0.4 ha); and R0.1 (0.1 unit/4 ha).

Smart Growth represents a policy that encourages more compact, high density development, with growth concentrated around existing urban peripheries. For this scenario, we re-allocated portions of the lower density classes from Business-as-usual into the more compact density classes, and created three new residential classes: R50 (50 units/0.4 ha), R10 (10 units/0.4 ha) and R0.5 (1 unit/0.8 ha). For example, 20% of R20 from Business as Usual was reallocated to R50 and 20% of R0.1 was reallocated to R1 (see Appendix SI for complete list of alterations). In addition, the attractiveness of the urban city centers was doubled for this scenario. The combined effect was to compel more compact and denser future urban growth along the edges of existing urban areas while reducing the amount of low-density housing that typically generates sprawl.

The Infill scenario was suggested by urban planners from the Association of Bay Area Governments. This scenario suggests that concentrating new growth is not sufficient, and that redevelopment, or the infilling and replacement of areas within existing city boundaries, is necessary to meet planning objectives, independent of conservation concerns. These objectives include reduction of greenhouse gases, reduction of commuting time, and lower emergency response time. For Infill, we used the residential classes from the Smart Growth scenario, but increased again the percentage of households in the high-density classes (Appendix S1). We ran the model twice for each county, the first time assigning all growth within existing urban areas. After assessing the number of residents and employees that would be displaced by infill development using Census 2000 data, we then ran the model again adding only the displaced persons outside of existing urban areas. The densities for housing and employment were again increased (Appendix S1). We then merged the two model runs for a final output allocation. In reality not all residents would be displaced, but the final model output simulates an urban footprint that incorporates a quantifiable proportion of the new population within the existing footprint.

### Open Space

There are 3723 Open Space areas within the extent of the historical landcover map (Figure 1). Spatial representation of these Open Space areas was acquired from the Bay Area Protected Areas Database [27]. We contacted local governments, managing agencies and environmental groups to obtain establishment dates for those properties. We divided the Open Space into areas established before 1940 (historical) and those established in and after 1941 (current). The cutoff date was selected to match the time frame of the historical landcover data set.

The BAUHG project used a four-year process to identify target conservation acquisition areas across the Bay Area that support a variety of conservation goals including protection of endemic species, ecological integrity and watershed functions [27]. We used their conservation targets within our study area to represent future regional conservation goals to 2050. This map of desired future open space was not used as a detractor in the urban growth models, to allow an assessment of where conflicts for space may arise.

### Data Analysis

For landcover change analyses, we first measured the extent of each landcover type, including urban extent, and calculated the area and percentage change, as increase or decrease from historic to current time. For future urban extents, we combined all urban classes in each UPlan model run to a single layer, and used that projection to measure change. Second, we repeated the same analysis for landcover within Open Space at each time frame, and calculated the representation of each landcover type (percentage protected in the available area of that landcover type at each time frame) and its change over time. We used the extent of urban as a co-occurring impact measure. Third, we overlaid the projected urban growth scenarios on the current landcover map and targeted future Open Space areas to assess their impacts under the different urban growth policies.

## Results

### Landcover Transitions

Within the 9275 km^2^ region, the largest landcover change was for Agriculture which went declined from 34.7% (3222 km^2^) to 5.8% (543 km^2^) in the region. Of the regional 28.8% lost (2679 km^2^), 15.5% (1440 km^2^) transitioned into Development. Overall, Development expanded almost 6 times in area (607%), from a historic 4.3% of the region to 24.1% of the region currently (Table 1).

Grasslands lost 257 km^2^ to Development, but 47 km^2^ transitioned to Chaparral, and 293 km^2^ into Hardwood. Hardwood lost 71 km^2^ to Development and 189 km^2^ to Chaparral, and also saw transition to Conifer-hardwood of 175 km^2^. Conifer-hardwood gained 39 km^2^ from Grassland, 56.8 km^2^ from Chaparral, and 175 km^2^ from Hardwood. Coastal Salt Marsh declined by 70 km^2^, including 37 km^2^ transitioned to Development (Appendix S2 for complete transition matrix).

### Urban Impacts

**Figure 2:**
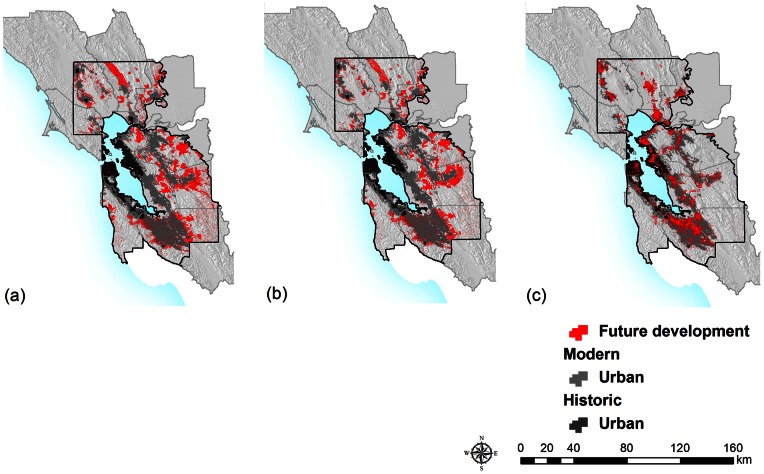
Urban Development trends and projections. (a) Future urban growth by 2050 Business-as-usual scenario (red); (b) Future urban growth by 2050 Smart Growth scenario and (c) Future urban growth by 2050 Infill scenario.

**Figure 3:**
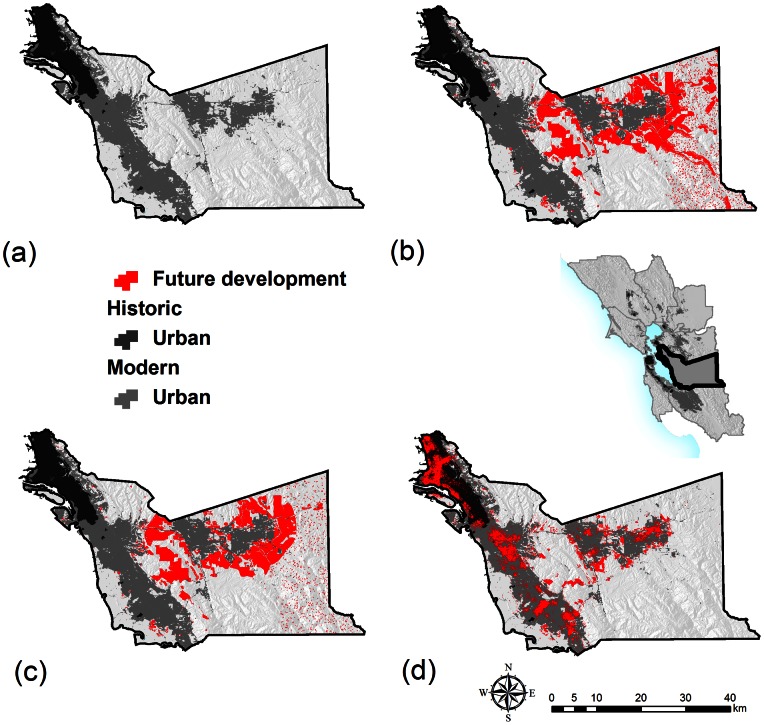
A detail of urban development trends and projections for Alameda County. (a) dark grey – Historic urban extents in 1940 and light grey – Present urban extent in 2011; (b) Future urban growth by 2050 Business-as-usual scenario (red); (c) Future urban growth by 2050 Smart Growth scenario and (d) Future urban growth by 2050 Infill scenario.

In the 1940s the urban extent was 4.3% of the region (396.5 km^2^). This expanded to 24.1% of the region (2239 km^2^) by current time. Under Business-as-usual, an additional 12.8% (1183 km^2^) of the study area becomes urban by 2050. Under Smart Growth, 11.3% (1044 km^2^) becomes urban, and under Infill, 8.7% (804 km^2^) becomes urban (Table 1; Figures 2, 3). The percent of new development located inside the existing urban footprint is 7.8% (92 km^2^) for the Business-as-usual, 8.4% (88 km^2^) for the Smart Growth, and 77.9% (626 km^2^) for the Infill scenario.

Of the current extents of Agriculture and Grassland an additional 52% is projected to be urbanized under Business-as-usual (169 km^2^ and 584 km^2^, respectively), compared with 43% under Smart Growth (134 km^2^ plus 536 km^2^, respectively), and 8% under Infill (21 km^2^ plus 107 km^2^, respectively). For natural landcover types, Hardwood would lose another 13.6% under Business-as-usual, 11.7% under Smart Growth, and 1.7% under Infill. For Conifer-hardwood, 5% is impacted under Business-as-usual, 4.2% under Smart Growth, and 0.6% for Infill. Riparian areas are also impacted by future growth, 11.7% under Business-as-usual, 11.7% under Smart Growth, and 5.6% under Infill (Table 1).

### Open Space Representation Analysis

Of the 3723 current Open Space properties, 2263 were dated, 516 cannot be dated because of the lack of information (110 km^2^, 4% of area covered by Open Space), and 944 remain to be dated (182 km^2^, 6%). By 1940, 3% (348.2 km^2^) of the study area was designated as Open Space, which grew to 20.2% (1873.4 km^2^) of the region today (Figures 2, 3). The future Open Space targets add an additional 11.1% (1038 km^2^) of the study area, leading to desired overall landscape protection of 35.1% of the region (BAUHG 2011).

Open Space created by 1940 included 116 km^2^ of Grassland, 87 km^2^ of Hardwood, and 43 km^2^ of Chaparral, and only 0.59 km^2^ of Riparian areas (Table 2). By 2010, protection of the same classes corresponds to an additional 586 km^2^ of Grassland, 447 km^2^ of Hardwood, and 158 km^2^ of Chaparral. The establishment of urban parks that contain buildings resulted in an increase in urban extents identified as Open Space, from 14 km^2^ to an additional 33 km^2^ in current time. Targeted future Open Space would protect an additional 395.3 km^2^ of Grassland, 222.4 km^2^ of Hardwood, and 128.7 km^2^ of Conifer-hardwood (Table 2).

**Table 2:** Landcover type representation in Open Space areas in the historic (1940), current (2006) and BAUHG future landscapes (2050).

	Open Space by 1940	Open Space by 2010	Future Open Space BAUHG
Agriculture	27	43	14
Grassland	116	586	395
Coastal Salt Marsh	3	64	11
Coastal Chaparral	34	79	30
Chaparral	43	158	127
Hardwood	87	447	222
Conifer-hardwood	9	258	129
Barren	2	14	5
Riparian	1	10	4
Wetland/Riparian	1	24	5
Water	12	159	19
Developed	14	33	77
Total	348	1873	1038

We excluded areas labeled water in both landcover maps. The totals refer to the study area extent and not to the full extent of Open Space in the Bay Area.

### Assessment of Future Threat to Current and Future Parks and Open Space

A total of 7.6% (79 km^2^) of targeted future Open Space is projected to be developed under Business-as-usual, 7.8% (81 km^2^) under Smart Growth, and 4.9% (51 km^2^) under the Infill scenario. Impacts to vegetation types and agriculture from projected future urban growth is essentially proportional to the policy, with Business-as-usual having the highest impact. Smart growth preserves 12% of each landcover type on average, relative to Business-as-usual, while Infill preserves 60.4%, on average (Table 1).

## Discussion

In the Bay Area, 75.8% of urban expansion over the last 70 years occurred on Agriculture and Grassland. This indicates that lands used for urban growth had already been converted to some level of human use (agriculture) at the beginning of our study. Our findings corroborate earlier analyses of a subset of our study area, where it was shown that pre-1940 land conversion to agriculture occurred on valley floors and impacted a variety of vegetation types [39], [40]. California agriculture, and grasslands and woodlands used in ranching retain some of the biological values of native landcover types [41], so likely the 1940 landscape was still widely useable by vertebrates and most ecosystem processes were functioning. But these landcover types, including extensive fruit orchards, ranch lands and row crops [19], [42], became the primary targets of urban development from 1940 to current time. This conversion from natural vegetation, to agriculture and ranch lands, to urban is a recognized sequence of development [43]. In this study we refer to agriculture and grasslands together because of the limited ability to thematically resolve these two landcover classes in the maps we had. Our transitions also show that 887 km^2^ of Agriculture transitioned to Grassland (Appendix S2). However, we are unaware of reports of retiring agriculture, and consider it likely that some ranch lands were included in the historic maps as Agriculture. Regionally, grasslands and agriculture together declined by 61%. While some Hardwood areas may also support ranching, as a whole these slightly decreased as well. Therefore, area available for agriculture and ranching combined declined. The potential consequences for both biodiversity and food security from this ongoing pattern should be of interest to regional planners.

Future urban extents were modeled to meet demand from a predicted increase of 3.07 million people. Most projected urban areas follow the same trajectory as observed from historic to modern times, by taking over Agriculture and Grassland. Hardwood was the third most threatened landcover type under predicted future development, with new impacts up to 13.6% of its current extent (Table 1). The future growth scenarios show how planning could influence very different futures in an already heavily modified landscape. The first finding was that even under the Business-as-usual scenario (the most extreme and least new planning required) there is enough space in the study region to accommodate most current Open Space goals and new urban growth. However, 79 km^2^ are still selected by both needs, and theewfore projections of development could be used by conservation organizations to prioritize lands for protection that are threatened under one or more development scenarios. Conservation actions have previously been undertaken in response to development pressure, which is a typical way conservation in developing regions may occur [44]. However, responding to emerging pressure from urban development by land acquisition is not an optimal approach for conservation planning, because it typically addresses near-term development pressure, and is therefore reactive. Reacting, rather than proactively developing conservation objectives with a longer planning timeframe, is limiting for conservationists because it can force conservation efforts to pay a premium for lands, and the consequences of failing to obtain the targeted lands can mean that they will imminently be developed. Alternatively, future conservation goals could also be included in urban growth planning to produce an alternative “green” scenario where urban growth is shunted away from important areas for conservation. In this study, the regional conservation plan, which we would call a ‘Greenprint’, proved essential for assessing projected impacts from urban growth across a 40-year time horizon.

Another key finding is that the Smart Growth scenario did not produce very different results from Business-as-usual, except for a reduction in extent of very low density residences (from 174 km^2^ to 80 km^2^, the speckle pattern in Figure 2). This exurban growth corresponds to low density rural housing units, which are recognized to be one of the most ecologically impacting forms of housing [45], and so Smart Growth does reduce ecological impacts from that stand point. However, given the interest in this approach [18], it is important to point out that simply denser new urban growth had little effect on reducing impacts to remaining Open Space, preserving only 12% of lands that would otherwise be developed. The implementation of the Infill scenario, effectively placing 62.9% of new urban growth inside urban boundaries, would have the effect of not developing between 57 and 60.4% of lands that would be developed under Smart Growth of Business-as-usual (Table 1, Appendix S1). For implementation, the Infill scenario would need an urban development policy that promotes redevelopment to the extent modeled.

The environmental footprint of an urban area includes all the areas required to supply food, water, construction materials and other supplies [19]. The historic spread of Bay Area urban development displaced food production that either caused new agricultural areas to be developed, used surplus production from existing agriculture (predominantly elsewhere), that benefited from increased production efficiency, or some combination of these alternatives. Urban spread into Agriculture and Grassland continues under the policy scenarios tested, with 3.9–22.8% of the remaining extents of these categories converted (Table 1). This urban growth will directly reduce remaining Agriculture and Grassland, with the assumption that the resources they provide will be displaced, but provided from other locations. Loss of these lands to urban development represents a reduction in the regional food security, which could be a factor in deciding which urban growth scenario is most appropriate. These results reinforce the need for an integrated assessment of competing land use demands in urban policy and point to the need for regional planning.

Several natural landcover types increased in extent from 1940 to current time, including Hardwood and Conifer-hardwood, which contribute ecologically and aesthetically to the region. This is likely due to the potentially lowered rates of fire [46], [47], [48], grazing [47], [48], and wood cutting [49] associated with the region’s conversion to natural gas for heating buidings [48], [50]. We also observed a 38.6% decline in the restricted and ecologically important Coastal Salt Marsh. While the rates of salt marsh loss vary by location, it is possible that this loss is due to a decrease in sediment inputs from the Sacramento River in the north [51], in addition to conversion by humans. It is also possible that the majority of the decline, 91 km^2^ into open water, is due either to historic sea level rise [52] or is an artifact of the classification system, or map accuracy.

The increases in natural landcover types are also due in part to an increase in extent of Open Space (2221 km^2^ added), as well as improved management for these types on both protected and unprotected lands. The Bay Area already had 348.2 km^2^ protected by the conclusion of the regional VTM surveys in 1937, thanks to forward-looking early conservationists such as Carolyn Livermore and John Muir [19]. Conservation efforts continued from that date to the present, with large increases in protected lands occurring in the 1960–1980s [19], and resulting in current open space protections for 24% of the study region. This trend of increasing the extent of protected areas is expected to continue with the guidance provided by the BAUHG assessment [27], which seeks further preservation of threatened agricultural, ranching and natural landcover areas. For example, 87 km^2^ of Hardwood was protected in 1940, experiencing a tremendous gain to a current protected extent of 554 km^2^, or 29.6% of the extent of current protected areas. Future conservation goals would extend Hardwood presence on protected lands another 222 km^2^.

This study focused on linking historic and future maps to create a temporally integrated assessment for a major metropolitan region. While we had good measures of spatial accuracy for input historic and contemporary landcover maps, the thematic accuracy of these maps is less certain. Both landcover maps had thematic limitations that needed to be addressed to be cross-comparable. The landcover maps form the basis for measures of change in urban and natural vegetation extents, and also for what vegetation types might be protected under future conservation goals, so a robust, if simplified classification is necessary. Greater confidence in the measured changes could be developed through cross-checking both landcover maps with aerial photographs (where available), but was beyond the scope of this study.

The Bay Area was a good region to apply our framework, because of the historical data available for trend assessment in the three areas measured: landcover, urban growth and open space. This is partially because of the historic landcover map, the region’s long and active conservation history, and the availability of a regional Open Space Greenprint. The historic trends provide perspective regarding future forecasting. Projected expansion of urban areas follows extensive historic growth. The historic preservation of Open Space is proposed to continue, with environmental groups and government agencies suggesting an increase to 35% of the study area [27]. Regionally, with limited additional area remaining to be allocated, the marginal Open Space gains represented under Smart Growth policy appear to be insufficient, and an Infill policy will better accommodate multiple needs for agriculture and ranching, for biodiversity preservation, and ecosystem function. Additionally, historic and continuing implementation of open space in the Bay Area contributes to its widely recognized high quality of life.

We propose that this approach, the aligning of several lines of evidence and the linking of historic trend to different projections of future condition, can be applied to other metropolitan areas. The framework can especially be useful to test differing scenarios of future growth with regards to how successful they may be in preserving Open Space needed for a wide variety of purposes. The resulting information is of broad interest for those interested in the sustainability of growing urban areas, particularly because regional perspectives help place ongoing dynamics in perspective. Potential implementers include city planners and their geographic information systems staff, landscape architects, researchers from academia, and conservation organizations. The approach also opens opportunities for collaboration between Open Space interest groups and land use or city planners. Criteria for the urban growth model runs can be decided in conjunction with city planners and model implementation can be done, for example, by the city planning, by regional parks and recreation departments, or by non-governmental organizations. A potential limiting factor is the availability of historic data. These data may exist for specific areas [53], [54] or may be reconstructed [55]. In most cases there exists at least satellite imagery, such as from the National Aeronautics and Space Administration Land-Cover Land Use Change program (http://lcluc.umd.edu/) that can be used to generate historic maps. A variety of urban growth models are available for application [56]. We used UPlan because it requires relatively low levels of parameterization, but various policies can be simulated with it [29], [32], [33].

## Supporting Information

Appendix S1 **This appendix contains the inputs for the three UPlan urban growth model scenarios presented in the paper.**
(DOCX)

Appendix S2 **This appendix contains the full transition table for the historic to current landcover type transitions.**
(DOCX)
