# Supplementary material for: Regional Assessment of Urban Impacts on Landcover and Open Space Finds a Smart Urban Growth Policy Performs Little Better than Business as Usual
Source: PLoS One. 2013 Jun 5;8(6):e65258. doi: 10.1371/journal.pone.0065258 (PMC3673918; doi:10.1371/journal.pone.0065258)
Supplement: Appendix S1 — This appendix contains the inputs for the three UPlan urban growth model scenarios presented in the paper. (DOCX) [file pone.0065258.s001.docx]

**Appendix SI.** UPlan parameters for three growth scenarios (Business as usual, Smart Growth, and Infill) and the residential, commercial and industrial land use classes used to parameterize the model.

The UPlan model works based on the following assumptions: (1) population growth can be converted into demand for land use by applying conversion factors to employment and households; (2) new urban expansion will conform to city and county general plans; (3) cells have different attraction weights because of accessibility to transportation and infrastructure; (4) some cells, such as lakes and streams, will not be developed; and (5) other cells, such as sensitive habitats and floodplains, will discourage new development, and can be assigned discouragement values.

UPlan considers five groups of attractors to development under the assumption that development occurs in areas that are attractive due to their proximity to existing urban areas and transportation facilities, such as freeway ramps. It is also assumed that the closer a vacant property is to an attraction, the more likely it will be developed in the future. The attractors are defined for the following land use classes: (1) Industrial, (2) High Density Commercial and Low Density Commercial; (3) High Density Residential (R50); (4) Medium Density Residential (R5, R10 and R20) and (5) Low Density Residential (R1, R.5 and R.1). The attractions for one land use group are not necessarily the same as those for another group, and the attractions for one land use group will have no impacts on the allocation of other land use types. Attractors considered are: highways, major roads, minor roads, city boundaries, and highway access ramps. The parameterization of the UPLan model scenarios used in this study are shown in Table S1.

In any scenario, there are areas where development cannot occur, called exclusions. Exclusions included features such as lakes and rivers, public open space, existing built-out urban areas, and other such features. Some features such as habitats, 100-year floodplains, and farmland might be developable at a high price. These features are called discouragements. Any features which will discourage development can be used as discouragements.

**Table S1.** Land use types used in each scenario (Business as usual – BAU, Smart Growth – SG, and Infill – IN), their description and percentage of houses according to US Census (2000) or classes created for future scenarios, scenarios where each class was used and the corresponding modifications to the base scenario (BAU) to create the Smart Growth and Infill scenarios.

| **UPlan Land Use Name** | **Description/Average Density** | **Percentage of houses** | **Scenarios Where Used** | **SG corresponding land use type in BAU** | **IN corresponding and use type in BAU** |
| --- | --- | --- | --- | --- | --- |
| Ind | Industry  55 m^2^/employee  0.23 FAR (Floor-Area-Ratio) |  | BAU, SG, IN* |  |  |
| CH | High-density Commercial  45 m^2^/employee  0.35 FAR (Floor-Area-Ratio) |  | BAU, SG, IN* |  |  |
| CL | Low-density Commercial |  | BAU, SG, IN* |  |  |
| R50 | Residential 50 (50 units/0.4ha) | 50+ unit apartments | SG, IN | 20% R20 | 60% R20 |
| R20 | Residential 20 (20 units/0.4ha) | 2 unit, 3-4 unit, 5-9 unit, 10-19 unit and 20-49 unit apartments | BAU, SG, IN | 20% R5 + 80% R20 | 40% R5 + 40% R20 |
| R10 | Residential 10 (10 units/0.4ha) | Category created for future scenario; 20% of the ratio for R5 from BAU, shifted to R10 for SG | SG, IN | 20% R5 | 60% R5 |
| R5 | Residential 5 (5 units/0.4ha) | Single family detached homes, <1 acre; single family attached homes, <1 acre | BAU, SG, IN | 60% R5 + 20% R1 | 100% R1 |
| R1 | Residential 1 (1 unit/0.4ha) | Single family detached homes, 1-9.9 acres; single family attached homes, 1-9.9 acres; and mobile homes, 1-9.9 acres | BAU, SG, IN | 80% R1 + 20% R.1 | 40% R.1 |
| R.5 | Residential 0.5 (1 unit/0.8ha lots) | Category created for future scenario; 20% of the ratio for R.1 from BAU, shifted to R.5 for SG | SG, IN | 20% R.1 | 40% R.1 |
| R.1 | Residential 0.1 (4ha lots) | Single family detached homes 10+ acres; single family attached homes, 10+ acres; Mobile homes, 10+ acres | BAU, SG, IN | 60% R.1 | 20% R.1 |

*The commercial and industrial classes for the Infill Scenario reduced the square footage/employee by 50%

### Demographic Data

- Base population: The population of the county in the year 2000, according to the U.S. Census. This figure was calculated by subtracting out the population living in institutions.
- Future Population: The population of each county in the year 2050, as projected by the Public Policy Institute of California.
- PPHH: Average persons per household for each county, according to the U.S. Census. PPHH is calculated by dividing the population (not including those living in institutions) by the number of households in the county.
  - Residential percentages: for each Residential type, the percentage of houses within that category. For R10, R5, R1, R.5 and R.1, categories created from a Special Tabulation by the U.S. Census cross-tabulated dwelling type (single family attached, single family detached, and mobile homes) by acreage (<1, 1-9.9, 10+) (Table S1).
- Employment percentages: the ratio of employees in one of three categories (Commercial High, Commercial Low, and Industry) to the total number of employees within the county (Census Transportation Planning Products 2000). See the separate table in Appendix C for CTPP industry to UPlan Industry crosswalk.
- Emp/HH: Average number of employees per household for each county, according to the U.S. Census, 2000. Population 16 years and over in labor force, males plus females, divided by the number of housing units.
- Average Square Footage per Employee, by Employment Type: for each employment type, the average number of square feet taken up by each employee. We used a statewide average for each county.
  - Industry: 56.9m^2^ (613 sq.ft.)/employee; From Arthur C. Nelson, 2004 (national average)
  - Commercial Low: 79.5m^2^ (856sq.ft.)/employee; from U.S. Energy Information Administration, 2003 Commercial Buildings Energy Consumption Survey, Revised June 2006, Table B.1, average for floors 1-3, factored by ratio of mean sq. ft. per worker for the Pacific states (.844).
  - Commercial High: 46.3m^2^ (498sq.ft.)/employee; from U.S. Energy Information Administration, 2003 Commercial Buildings Energy Consumption Survey, Revised June 2006, Table B.1, average for floors 4-9 and 10 or more floors, factored by ratio of mean sq. ft. per worker for the Pacific states (.844).
- FAR: Floor-Area Ratio: proportion formed by a building’s built floor area (for all floors) divided by the area of its land parcel.  For example, a one-story building with a floor plan length and width of 30.4x30.4 m (100 feet by 100 feet) on the same size land parcel would have an FAR. of 1.0.
  - Industry: 0.23
  - Commercial Low: 0.15
  - Commercial High: 0.35

### Spatial Data

All spatial data were converted to Albers Equal Area NAD83 Prior to use in the modeling environment.

#### Base Layers

- County boundary: extent of each county, Department of Conservation, 2007
- General Plan: General plan of each county, modified to a statewide set of types.

#### Attractors

- Highways: Tele Atlas North America, Inc., ESRI, 2005; FCC = A2
- Major Roads: Tele Atlas North America, Inc., ESRI, 2005; FCC = A3
- Minor Roads: Tele Atlas North America, Inc., ESRI, 2005; FCC = A4
- Ramps: Tele Atlas North America, Inc., ESRI, 2005; FCC = A63
- Places: City boundaries, Census 2000
- Blocks with Growth: U.S. Census Blocks with a population increase from 1990-2000

#### Discouragers

- CNDDB: California Natural Diversity Database, Department of Fish and Game, downloaded September, 2010.
- Floodplains: FEMA Q3 flood zones, downloaded 2005
- Vernal Pools: Holland, 2005 (revised 2009)
- Wetlands: National Wetlands Inventory, January 2010 (removed ty = “riverine”)

#### Masks

- CPAD: California’s Protected Areas Database, GreenInfo Network, 2010. Units Fee. San Francisco, CA.
- Existing Urban: California Augmented Multisource Landcover Map (CAML), CAIN at ICE, U.C. Davis, 2006.

**References**

Nelson AC (2004) Toward a new metropolis: the opportunity to rebuild America, Metropolitan Policy Program, Brookings Institution.

Department of Conservation (DOC) and the California Department of Forestry and Fire Protection (CAL FIRE) (2007) County Boundaries (1:24K). http://projects.atlas.ca.gov/frs/?group_id=41.

Tele Atlas North America, Inc., ESRI, (2005)

U.S. Census Bureau; Census 2000. 2000. U.S. Census Bureau, Washington, D.C.
