# Supplementary material for: Regional Assessment of Urban Impacts on Landcover and Open Space Finds a Smart Urban Growth Policy Performs Little Better than Business as Usual
Source: PLoS One. 2013 Jun 5;8(6):e65258. doi: 10.1371/journal.pone.0065258 (PMC3673918; doi:10.1371/journal.pone.0065258)
Supplement: Appendix S2 — This appendix contains the full transition table for the historic to current landcover type transitions. (DOCX) [file pone.0065258.s002.docx]

**Appendix SII.**

**Table S2.** Historic to current landcover transitions. Rows represent historic landcover classes and columns represent current landcover classes. Values are in km^2^.

| Landcover Class | Historic Total | Agriculture | Grassland | Coastal Salt Marsh | Coastal Chaparral | Chaparral | Hardwood |
| --- | --- | --- | --- | --- | --- | --- | --- |
| **Agriculture** | 3222 | 429 | 887 | 14 | 44 | 34 | 215 |
| **Grassland** | 2161 | 45 | 1384 | 6 | 46 | 47 | 293 |
| **Coastal Salt Marsh** | 182 | 0 | 15 | 30 | 0 | 0 | 1 |
| **Coastal Chaparral** | 270 | 1 | 40 | 0 | 96 | 38 | 38 |
| **Chaparral** | 673 | 9 | 39 | 0 | 19 | 346 | 191 |
| **Hardwood** | 1796 | 35 | 334 | 1 | 14 | 189 | 957 |
| **Conifer-hardwood** | 246 | 1 | 6 | 0 | 3 | 8 | 16 |
| **Barren** | 46 | 0 | 3 | 7 | 1 | 0 | 1 |
| **Riparian** | 2 | 0 | 0 | 0 | 0 | 0 | 0 |
| **Wetland/Riparian** | 14 | 1 | 3 | 0 | 0 | 0 | 3 |
| **Water** | 267 | 22 | 35 | 53 | 1 | 2 | 5 |
| **Developed** | 396 | 0 | 17 | 1 | 4 | 0 | 10 |
| **Current Total** | 9275 | 543 | 2763 | 111 | 229 | 667 | 1731 |

**Table S2 (cont.).** Historic to current landcover transitions. Rows represent historic landcover classes and columns represent current landcover classes. Values are in km^2^.

| Landcover Class | Conifer-hardwood | Barren | Riparian | Wetland/Riparian | Water | Developed |
| --- | --- | --- | --- | --- | --- | --- |
| **Agriculture** | 68 | 14 | 22 | 13 | 43 | 1440 |
| **Grassland** | 39 | 6 | 5 | 6 | 27 | 257 |
| **Coastal Salt Marsh** | 0 | 0 | 0 | 7 | 91 | 37 |
| **Coastal Chaparral** | 42 | 2 | 2 | 0 | 1 | 10 |
| **Chaparral** | 57 | 2 | 1 | 0 | 1 | 7 |
| **Hardwood** | 175 | 3 | 7 | 0 | 10 | 71 |
| **Conifer-hardwood** | 210 | 0 | 0 | 0 | 0 | 0 |
| **Barren** | 2 | 0 | 0 | 2 | 23 | 5 |
| **Riparian** | 0 | 0 | 0 | 0 | 0 | 0 |
| **Wetland/Riparian** | 0 | 0 | 1 | 0 | 1 | 4 |
| **Water** | 3 | 13 | 0 | 14 | 61 | 56 |
| **Developed** | 10 | 0 | 1 | 0 | 3 | 351 |
| **Current Total** | 606 | 42 | 41 | 42 | 262 | 2239 |
